# The core of performance in adolescent cricket pace bowlers: Trunk muscle stability, maybe, but not strength-endurance and thickness

**DOI:** 10.17159/2078-516X/2022/v34i1a12521

**Published:** 2022-01-01

**Authors:** FM Olivier, B Olivier, N Mnguni

**Affiliations:** 1 Wits Cricket Research Hub for Science, Medicine and Rehabilitation, School of Therapeutic Sciences, Faculty of Health Sciences, University of the Witwatersrand; 2 Department of Physiotherapy, School of Therapeutic Sciences, Faculty of Health Sciences, University of the Witwatersrand

**Keywords:** fast bowling, core muscles, ball release speed, accuracy

## Abstract

**Background:**

The trunk connects the upper and lower limbs and transfers energy during movement. Exploring the role of the trunk muscles in bowling performance affords us the opportunity to uncover potential mechanisms to improve bowling performance.

**Objectives:**

To investigate the association between bowling performance and trunk muscle stability, strength-endurance and thickness in adolescent pace bowlers.

**Methods:**

Adolescent pace bowlers participated in this cross-sectional study. Trunk muscle stability was measured using Sahrmann’s Stability Scale, strength-endurance using the Bourbon Trunk Muscle Strength Test and thickness of the abdominal wall and lumbar multifidus muscles using ultrasound imaging.

**Results:**

Forty-six pace bowlers with a mean age of 15.9 ±1.2 years participated. The average ball release speed was 109.2±11.8 km.h^−1^. This measurement was higher in level four of stability than in level two (mean difference 22.2 ± SD 6.8 km.h^−1^; p= .018). No link between ball release speed and strength-endurance could be found. Multiple correlations of moderate strength (r > 0.4) exist between ball release speed and absolute trunk muscle thickness with height and weight as confounding factors. The relationship between accuracy and the trunk muscle variables investigated in this study is weak.

**Conclusion:**

Bowlers with better trunk muscle stability bowled faster than those with a lower level of trunk stability, irrespective of their age, height and weight. Trunk muscle thickness correlated with ball release speed; however, confounding factors such as height and weight play a role and therefore, findings need to be interpreted with caution.

In cricket, the pace bowler aims to outsmart the batsman on strike with a good quality delivery performed at maximum ball release speed, with perfect accuracy, and a good line and length. The bowling action consists of four phases: the run-up phase, the pre-delivery (or gather) phase, the delivery phase, and the follow-through phase, with unique trunk movements contributing to each phase. The pre-delivery phase requires the trunk to hyperextend on back foot landing. As the ball is being delivered, the trunk moves into flexion (lateral flexion) away from the delivery arm, and rotates towards the non-delivery arm. The repetitive execution of the bowling action activates the trunk muscles in the same asymmetrical manner with each delivery, potentially resulting in this particular pattern of muscle hypertrophy.^[1, 2]^

The topic of trunk musculature is frequently raised when sport and performance are discussed. Earlier research from Hodges and Richardson^[3]^ shows that the transversus abdominis activates before the trunk starts moving. This implies that the transversus abdominis acts as a trunk stabiliser when power is produced by the extremities. Akuthota and Nadler^[4]^ further explain that when the external oblique, internal oblique and transversus abdominis contract, the intra-abdominal pressure increases, tensioning the thoraco-lumbar fascia. The thoraco-lumbar fascia connects to the upper and lower limbs posteriorly and by incorporating the abdominal fascia anteriorly and the oblique muscles laterally, a corset is formed around the abdomen, so increasing the intra-abdominal pressure.

Opposing views exist regarding the role of trunk muscle stability and strength-endurance in athletic performance as investigated in sports other than cricket. An eight-week core endurance programme improved some aspects of core stability in rowers but did not affect their functional performance.^[5]^ Similarly, after six weeks of core conditioning training in male runners, core stability improved but running economy remained unchanged.^[6]^ In contrast, core stability training has been shown to improve sports performance measures in other studies ^[7, 8]^. Throwing velocity improved after handball players underwent 10 weeks of core training.^[7]^ In a cross-sectional study amongst action cricket fast and fast-medium bowlers, a relationship was found between good core stability and a high ball release speed; however, trunk muscle strength-endurance, thickness and the accuracy of deliveries were not measured.^[8]^

The thickness and symmetry of trunk muscles between dominant and non-dominant sides in injured and uninjured pace bowlers has been investigated by means of ultrasound. In bowlers with no pain, the abdominal wall of the non-dominant side was thicker than that of the dominant side.^[1, 2]^ There was no difference in side-to-side thickness of the transversus abdominis.^[2]^ In another study, the cross-sectional area of the lumbar multifidus was symmetrical in injury-free bowlers, but smaller on the non-dominant side in those who had sustained an injury during the season.^[9]^ Different patterns of muscle morphometry exist in adolescent pace bowlers, depending on whether or not they have pain during movement. Asymmetry of the trunk muscles and the effect of injury on the trunk muscle morphometry in the prediction of potential injury has been well described.^[1, 9]^ However, compared to other sporting codes, studies investigating the relationship between pace bowling performance and trunk muscle stability, information on strength-endurance, and trunk muscle thickness are lacking in the literature. Pace bowlers may contribute greatly to a team’s success when bowling accurately, at the right speed, and taking wickets or putting the opposition’s run-rate under pressure. Therefore, this study aims to investigate the association between bowling performance, namely ball release speed and bowling accuracy, and trunk muscle stability, trunk muscle strength-endurance and trunk muscle thickness in adolescent pace bowlers.

## Methods

### Study design, study setting and participants

This observational cross-sectional study included pace bowlers between the ages of 13 and 18 years from their school’s A-teams. Directors of cricket at four secondary schools had their A-team coaches nominate all pace bowlers to be invited to take part in the study. Only male participants were included due to the limited number of female players, and to create a homogenous group. Participants with previous upper or lower limb surgery or extensive lower back interventions, e.g. spinal facet infiltrations, were excluded from the study.

Data collection took place in the outdoor cricket nets of the participating schools. This study was adapted from a research report conducted to fulfil requirements for the degree of Master in Physiotherapy,^[10]^ thus ethical clearance was obtained from the Human Ethics Research Committee of the University of the Witwatersrand, and both consent and assent was signed by parents and participants respectively. The sample size was calculated using Cohen’s default interpretations, using G Power 3.1.9.2 and a two-tailed design. The power of 0.8 with a medium to large effect size (0.4) was calculated as 44 participants. The resulting critical t value was 2.02, with a power value of 0.955.

### Protocol

**Fig. 1:**
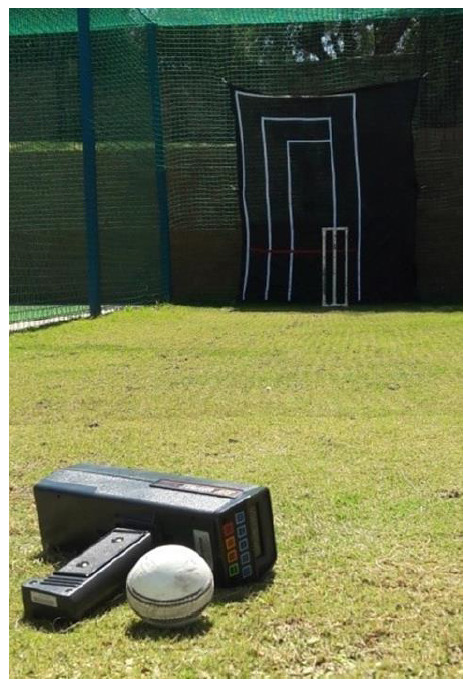
Bowling accuracy target

The participants were instructed to bowl six match pace deliveries, one ball following the other with approximately one minute in between deliveries, aiming for the top of the wicket on the off-side as if they were bowling to a right-handed batter. Each participant was allowed three practice balls to familiarise themselves with the target. Ball release speed was measured using a hand-held radar speed gun (Stalker, ATS, Texas). The radar speed gun was positioned approximately five metres behind the point of ball release, as closely in line with the bowler’s upper limb trajectory as possible. The average ball release speed was used in the analysis. Bowling accuracy was measured using a black shade cloth target with scoring zones sewn onto it in white and a horizontal line in red, 50 centimetres off the ground. A maximum of 100 points was scored if the ball made contact in the zone of the off-stump, 50 and 25 points in adjacent zones and zero if the target was missed altogether (Figure 1).^[11]^ The median accuracy score was used in the analysis.

**Fig. 2:**
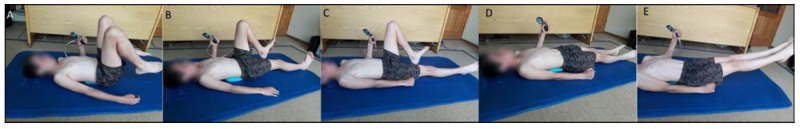
Sahrmann Stability Scale: A) Level 1; B) Level 2; C) Level 3; D) Level 4; E) Level 5

The Sahrmann Stability Scale^[12]^ was used to assess trunk muscle stability. A Chattanooga pressure biofeedback (Encore Medical, Australia) was inflated to 40 mm Hg and positioned in the lumbar lordosis with the participant in supine crook-lying ^[13]^. The participant was instructed to maintain the “draw-in manoeuvre” throughout a level, not allowing the spine to move, which would result in a pressure deviation of more than 10 mm Hg on the pressure biofeedback – an indication of spinal stability lost. The score achieved was the level successfully passed on a scale of one-five, where after the legs were added acting as levers to increase the level of difficulty (Figure 2).

The Bourbon Trunk Muscle Strength Test (TMS) ^[14]^ was used to assess trunk muscle strength-endurance. Time was measured in seconds at the point of failure for the particular muscle chain. The ventral, lateral left and right, and dorsal trunk muscle chain tests were performed in randomised order with a 10 minute recovery time allowed between tests.

Muscle thickness of the transversus abdominis, external oblique and internal oblique was measured at rest, and during the abdominal drawing-in manoeuvre. The lumbar multifidus muscle at the L4, 5 facet joint was measured at rest using ultrasound imaging. Measurements were recorded in millimetres with on-screen callipers - from the middle of the one fascia layer to the middle of the next layer - using a DP-6600 Digital ultrasonic imaging system® (Shenzhen Mindray Bio-medical Electronics Co., Ltd, China) with a 5MHz curvilinear transducer and a large footprint (≥60mm).

### Data reduction

The following calculations were done:

- Percentage change (a percentage of muscle thickness at rest) = (muscle activated − muscle at rest) ÷ muscle at rest × 100^[2]^
- Contraction ratio (the ratio of the contracted muscle thickness to the muscle thickness at rest) = muscle thickness contracted/muscle thickness at rest^[15]^
- TA preferential activation ratio (difference in the TA proportion of the total lateral abdominal muscle thickness in going from the relaxed to the contracted state) = (TA contracted/TA + IO + EO contracted) – (TA at rest/TA + IO + EO at rest)^[15]^
- Relative thickness (a percentage of the total thickness of all three muscles together) = EOrest/(EOrest + IOrest + TArest) x100^[15]^
- Relative thickness (a percentage of the total thickness of all three muscles together) = EOcont/(EOcont + IOcont + TAcont) ^[15]^
- Percentage difference (between sides) = [(largest/smallest value x 100) − 100] ^[9, 16]^

Where EO is external oblique; IO is internal oblique; TA is transversus abdominis

### Statistical analysis

Data were analysed using IBM SPSS Statistics for Windows (version 26.0. Armonk, New York, USA). A Pearson’s correlation was conducted for all parametric data, and a Spearman’s Rank order correlation was conducted for ordinal or non-parametric data. A value of 1 or −1 for the Pearson’s (r) and Spearman’s (ρ) correlation was considered to be a *perfect* association, positive and negative values above 0.70 were considered to be *strong* correlations, those between 0.40 and 0.70 were *moderate* correlations, and those below 0.40 were *weak* correlations. Multivariate analyses could not be performed due to the size of the dataset as well as a high level of multicollinearity among the independent variables. A one-way analysis of variance (ANOVA) followed by a Tukey post hoc test were conducted to determine whether there is a difference of means in average ball release speed among five levels of stability. Spearman’s Rank order correlation was conducted to determine the relationship between accuracy and ball release speed, trunk muscle stability, muscle strength-endurance, and muscle thickness. A Pearson’s correlation was conducted to assess the relationship between average ball release speed and muscle strength-endurance, as well as muscle thickness. The association between age and height, and weight with absolute trunk muscle thickness was determined using both the Pearson’s and Spearman’s Rank order correlation as appropriate for the distribution of the data. Ordinal logistic regression was used to explore the association between age, height and weight on the likelihood of a higher level of stability using the Wald test for parameter and model fit.

## Results

Forty-six pace bowlers with a mean age of 15.9 years (standard deviation (SD)=1.2; median= 16.0; range=14–18 years), a mean height of 173.9 cm (SD=8.0; median=174.0; range=157.0–193.0 cm) and mean weight of 65.0 kg (SD=16.8; median=62.5; range=43.0–140.0 kg) participated. Forty-two (91%) were right-handed and four (9%) were left-handed bowlers.

### Ball release speed and accuracy

The participants bowled at a mean of 109.2 km.h^−1^ (SD=11.8; median=105.0; range=85.5–128.9 km.h^−1^) and a median accuracy score of 70 percent (range=56–87 %) was attained. There was no association between ball release speed and accuracy (ρ= −.08; p= .586).

### Ball release speed, bowling accuracy, age, height and weight

Ball release speed was moderately associated with age (r= .43, p= .003) and height (r= .49, p= .001), and weakly associated with weight (r= .39, p= .007). However, there was no association between bowling accuracy and age (ρ= .04, p= .770), height (ρ = .06, p= .686) or weight (ρ= .09, p= .551).

### Ball release speed, accuracy and trunk muscle stability

The median value for trunk muscle stability was three (range=one-five) on the Sahrmann Stability Scale.

There was a difference between levels of performance on the Sahrmann Stability Scale as determined by one-way ANOVA (F(4,41)= 3.473; p= .011) (Table 1). A Tukey post hoc test revealed that the average ball release speed was higher in Level four of stability than in Level two (mean difference 22.2 ± 9.6 km.h^−1^; p= .018).

There were no differences in ball release speed among the other levels of stability. There was no relationship between bowling accuracy and trunk stability (ρ= −.011; p= .940). Ordinal logistic regression was performed to determine the effect of age, height and weight on the likelihood of a higher level of stability. The model explained 4.6% (Nagelkerke R^2^) of the variance in levels of stability. The logistic model was not a good fit (Χ^2^(3)= 2.025; p= .567) and none of the predictor variables in the ordinal regression analysis were found to contribute to the model [age: Wald= 1.014, p= .314; height: Wald= .056, p= .495; weight: Wald= .897, p= .344].

### Ball release speed, accuracy and trunk muscle strength-endurance

Trunk muscle strength-endurance is shown in Table 2. There was no relationship between either ball release speed or accuracy, and strength-endurance.

### Ball release speed, accuracy and trunk muscle thickness

Multiple correlations of moderate strength (>0.4) exist between ball release speed and absolute trunk muscle thickness as measured at rest and in a contracted state (Table 3). To determine the role of age, height and weight in absolute trunk muscle thickness, correlations between these variables were determined and can be found in the Online Supplementary Material Tables 1, 2 and 3. Moderate correlations exist between age and two of the absolute trunk muscle thickness-related variables, with 16 weak correlations, while between height and absolute trunk muscle thickness, 13 of the 18 variables showed moderate correlations. Three strong, 13 moderate and two weak correlations were found between weight and absolute trunk muscle thickness.

No relationship seemed to exist between ball release speed and trunk muscle thickness-related calculations, such as percentage change, contraction ratio, relative thickness at rest or relative thickness when contracted (Table 4). The highest correlation was that of the contraction ratio between the thickness of the non-dominant internal and external oblique when contracted and the same muscles when at rest. However, this correlation can be interpreted as weak (r=.25; p= .028).

Some weak correlations were determined between bowling accuracy and absolute muscle thickness (Online Supplementary Material – Table 4), as well as between bowling accuracy and the derivatives of muscle thickness (Online Supplementary Material – Table 5). The highest of these was a correlation between the relative thickness of the dominant transversus abdominis, which is expressed as a percentage of total thickness of all three muscles together, and bowling accuracy (r= .40; p= .006).

## Discussion

The findings from this research study, a first of its kind, answered a valuable research question in our quest to uncover the exact mechanisms behind bowling performance. The findings from this paper form a basis for future research, where mechanisms related to trunk muscle stability, strength-endurance and thickness, can be explored further.

In this study, adolescent pace bowlers with better trunk muscle stability bowled faster than those with a lower level of trunk stability, irrespective of their age, height and weight. Hilligan^[8]^ reported a good relationship between ball release speed and trunk muscle stability in adult (18–35 years old) male indoor pace bowlers, although the participants in his study were older and performed slightly different stability tests. Portus et al.^[11]^ could not correlate trunk muscle stability to bowling performance (ball release speed and accuracy), but they found that the bowlers who scored higher on the trunk muscle stability test were also bowling with a slightly more bent knee at front foot impact. Therefore, bowlers landing on a bent knee are believed to have possibly adapted and improved their trunk muscle stability, enabling them to use the trunk as a rigid lever to generate ball release speed, instead of an extended knee. An extended knee angle has been associated with an increase in ball release speed, whereas a slightly flexed knee is recommended to prevent injury due to a reduction in load placed on the lumbar spine – a trade-off that is difficult to negotiate for any bowler. Therefore, it seems like bowling with a slightly flexed knee will reduce lumbar loads and increase trunk muscle stability, which will allow a bowler to bowl faster, despite bowling with a flexed knee. However, the exact mechanism is unclear, and causality cannot be assumed.

There was no relationship between ball release speed and trunk muscle strength-endurance, highlighting the potential differences in constructs tested using the Bourbon TMS, namely the amount of time a player can hold a certain position vs the functional demands of the pace bowling action. Also, none of the variables influenced players’ accuracy scores, which leaves us with the impression that accuracy is not dependent on trunk muscle stability, strength-endurance or thickness, but that other mechanisms are at play.

There seems to be an overall moderate correlation between ball release speed and absolute trunk muscle thickness, which means those who bowl faster have thicker trunk muscles. Although it is very tempting to recommend that trunk muscle thickness be increased to increase ball release speed, when analysing the role of age, height and weight, confounding relationships became apparent. There seems to be a pertinent relationship between weight and absolute muscle thickness, where the heavier bowlers had thicker muscles. However, there is a weak correlation between ball release speed and weight, which means those who bowled faster were not necessarily heavier. Age and height were moderately correlated with ball release speed, although only height showed a moderate relationship with absolute trunk muscle thickness. This points us to the complexity and the importance of considering confounding factors in our methodological planning and statistical analysis.

In this study, no regression analysis could be done due to multi-collinearity between variables. Considering these findings, results should be interpreted with caution, and findings may indicate pure associations while a cause-effect relationship should not be assumed. Future studies should include larger sample sizes which will allow for sub-group analysis and thus confounding variables can be eliminated. The inclusion of female pace bowlers, as well as an exploration of the role of the lumbo-pelvic-femoral complex and growth spurts on bowling performance in future research projects is strongly advised. Longitudinal studies are recommended to explore causality, something that was not possible in this study considering its cross-sectional nature. In addition, future intervention studies can explore the effectiveness of a trunk muscle strengthening programme on ball release speed while considering age, height and weight as confounders. It is possible that the shorter, lighter bowlers could possibly benefit from specific trunk muscle pattern strengthening to improve ball release speed, because a muscle should become thicker once it is strengthened. Against the results of the current study, this could then benefit the bowler in terms of ball release speed. However, the findings from this study cannot support such a recommendation in clinical practice.

## Conclusion

Adolescent pace bowlers with better trunk muscle stability bowled faster than those with a lower level of trunk stability, irrespective of their age, height and weight. Trunk muscle thickness correlated with ball release speed, however, confounding factors such as height and weight play a role and therefore this study’s findings need to be interpreted with caution and future research is required. Bowling accuracy was not linked to trunk muscle stability, strength-endurance or thickness, and other mechanisms are clearly at play.

## Supplementary Information



## Figures and Tables

**Table 1:** The association between ball release speed and trunk muscle stability (n=46)

Descriptive		One-way ANOVA Results					
Trunk muscle stability	Ball release speed (km.h^−1^)		Sum of square	Mean square	Degree of freedom	F-statistic	p-value
Level 1	106.4 (10.2)	**Between groups**	1607.6	401.9	4	3.473	.011
Level 2	99.7 (10.4)						
Level 3	105.5 (10.3)						

Level 4	121.9 (6.1)	**Within groups**	4402.7	107.4	41		
Level 5	118.22 (13.98)						

Data expressed as mean (SD) unless indicated otherwise.

**Table 2:** The association between strength-endurance and accuracy and average ball release speed (n=46)

	Strength-endurance (seconds)		Accuracy			Ball release speed	

Trunk muscle chain	Mean (SD)	Median (Range)	ρ	p-value	r	ρ	p-value
Dorsal	69.54 (23.10)	67.50 (25 – 130)	−.248	.096	-	.209	.164
Ventral	70.09 (27.91)	63.50 (23 – 131)	−.247	.098	.153	-	.155
Non-dominant lateral	48.07 (16.83)	45 (23 – 131)	−.034	.821	-	.013	.930
Dominant lateral	49.46 (16.20)	47.50 (16 – 89)	.125	.407	−.070	-	.322

r, Pearson’s correlation performed for parametric data; ρ, Spearman’s Rank order correlation performed for non-parametric data.

**Table 3:** The relationship between ball release speed and absolute trunk muscle thickness at rest and when contracted (n=46)

	At rest			Contracted		

Trunk muscle	r	ρ	p-value	r	ρ	p-value
Non-dominant EO	.02	-	.882	-	.22	.142
Dominant EO	.08	-	.602	.10	-	.519

Non-dominant IO	-	.33	.025	-	.45	.002
Dominant IO	.25	-	.091	-	.34	.020

Non-dominant TA	-	.53	<.001	-	.42	.004
Dominant TA	.54	-	<.001	-	.46	.001

Non-dominant ABD	.33	-	.026	-	.54	<.001
Dominant ABD	.32	-	.030	-	.40	.006

Non-dominant Multifidi	-	.4	.006	-	-	-
Dominant Multifidi	.20	-	.186	-	-	-

r, Pearson’s correlation performed for parametric data; ρ, Spearman’s Rank order correlation performed for non-parametric data; EO, external oblique; IO, internal oblique; TA, transversus abdominis; ABD, abdominal wall (EO+IO+TA).

**Table 4:** The relationship between ball release speed and derivatives of absolute trunk muscle thickness (n=46)

	r	ρ	p-value
**Percentage difference**			
EO at rest	.14	-	.176
IO at rest	.23	-	.062
TA at rest	.08	-	.303
ABD at rest	.11	-	.224
EO contracted	.02	-	.458
IO contracted	.11	-	.225
TA contracted	.05	-	.365
ABD contracted	.04	-	.404
Multifidi at rest	.10	-	.252

**Percentage change**			
Non-dominant EO	.24	-	.055
Dominant EO	-	.01	.952
Non-dominant IO	.19	-	.106
Dominant IO	.17	-	.124
Dominant TA	-	−.09	.561
Non-dominant TA	-	−.08	.618

**Contraction ratio**			
Non-dominant EO	.24	-	.055
Dominant EO	-	.01	.952
Non-dominant IO	.19	-	.106
Dominant IO	.17	-	.124
Non-dominant TA	-	−.08	.618
Dominant TA	-	−.09	.561
Non-dominant EOIO	.25	-	.028
Dominant EOIO	.16	-	.146

**Relative thickness at rest**			
Non-dominant EO	-	−.28	.058
Non-dominant IO	-	.18	.225
Non-dominant TA	-	.19	.215
Dominant EO	-	−.23	.122
Dominant IO	-	.01	.967
Dominant TA	-	.40	.006

**Relative thickness contracted**			
Non-dominant EO	−.06	-	.339
Non-dominant IO	.13	-	.187
Non-dominant TA	-	−.14	.370
Dominant EO	−.20	-	.094
Dominant IO	.05	-	.377
Dominant TA	-	.12	.412

r, Pearson’s correlation performed for parametric data; ρ, Spearman’s Rank order correlation performed for non-parametric data; EO, external oblique; IO, internal oblique; TA, transversus abdominis; ABD, abdominal wall (EO+IO+TA).
