# Supplementary material for: The core of performance in adolescent cricket pace bowlers: Trunk muscle stability, maybe, but not strength-endurance and thickness
Source: S Afr J Sports Med. 2022 Jan 1;34(1):v34i1a12521. doi: 10.17159/2078-516X/2022/v34i1a12521 (PMC9924598; doi:10.17159/2078-516X/2022/v34i1a12521)
Supplement: Supplementary file 1 [file 2078-516X-34-v34i1a12521-s001.pdf]

The core of performance in adolescent cricket pace bowlers: Trunk muscle stability, maybe, but not strength-endurance and thickness

Supplementary material – Table 1. The association between age and absolute trunk muscle thickness

	r	ϱ	p-value
**At rest**			
Non-dominant EO	.06	-	.686
Dominant EO	.13	-	.403
Non-dominant IO	-	.04	.769
Dominant IO	.25	-	.099
Non-dominant TA	-	.17	.274
Dominant TA	.19	-	.201
Non-dominant ABD	.14	-	.368
Dominant ABD	.24	-	.110
Non-dominant Multifidi	-	.38	.010
Dominant Multifidi	.18	-	.237
**Contracted**			
Non-dominant EO	-	.25	.093
Dominant EO	.23	-	.122
Non-dominant IO	-	.40	.006
Dominant IO	-	.35	.018
Non-dominant TA	-	.26	.082
Dominant TA	-	.29	.054
Non-dominant ABD	-	.45	.002
Dominant ABD	-	.31	.034

r, Pearson's correlation performed for parametric data; ϱ, Spearman's Rank order correlation performed for non-parametric data; EO, external oblique; IO, internal oblique; TA, transversus abdominis; ABD, abdominal wall

Supplementary material – Table 2. The association between height and absolute trunk muscle thickness

	r^{+}	$\varrho^{\#}$	p-value
**At rest**			
Non-dominant EO	.26	-	.078
Dominant EO	.40	-	.006
Non-dominant IO	-	.26	.088
Dominant IO	.45	-	.002
Non-dominant TA	-	.46	.001
Dominant TA	.55	-	<.001
Non-dominant ABD	.40	-	.002
Dominant ABD	.56	-	<.001
Non-dominant Multifidi	-	.59	<.001
Dominant Multifidi	.46	-	.001
**Contracted**			
Non-dominant EO	-	.30	.040
Dominant EO	.39	-	.007
Non-dominant IO	-	.43	.003
Dominant IO	-	.52	<.001
Non-dominant TA	-	.46	.001
Dominant TA	-	.51	<.001
Non-dominant ABD	-	.56	<.001
Dominant ABD	-	.58	<.001

r, Pearson's correlation performed for parametric data; ϱ, Spearman's Rank order correlation performed for non-parametric data; EO, external oblique; IO, internal oblique; TA, transversus abdominis; ABD, abdominal wall

Supplementary material – Table 3. The association between weight and absolute trunk muscle thickness

	r	ϱ	p-value
**At rest**			
Non-dominant EO	.33	-	.025
Dominant EO	.59	-	<.001
Non-dominant IO	-	.34	.022
Dominant IO	.62	-	<.001
Non-dominant TA	-	.63	<.001
Dominant TA	.66	-	<.001
Non-dominant ABD	.44	-	.002
Dominant ABD	.76	-	<.001
Non-dominant Multifidi	-	.73	<.001
Dominant Multifidi	.61	-	<.001
**Contracted**			
Non-dominant EO	-	.46	.001
Dominant EO	.70	-	<.001
Non-dominant IO	-	.43	.003
Dominant IO	-	.49	.001
Non-dominant TA	-	.56	<.001
Dominant TA	-	.57	<.001
Non-dominant ABD	-	.63	<.001
Dominant ABD	-	.67	<.001

r, Pearson's correlation performed for parametric data; ϱ, Spearman's Rank order correlation performed for non-parametric data; EO, external oblique; IO, internal oblique; TA, transversus abdominis; ABD, abdominal wall

Supplementary material – Table 4. The relationship between bowling accuracy and absolute muscle thickness

	ϱ	p-value
**At rest**		
Non-dominant EO	.08	.596
Dominant EO	.09	.537
Non-dominant IO	.02	.905
Dominant IO	-.22	.141
Non-dominant TA	-.03	.826
Dominant TA	-.11	.457
Non-dominant ABD	.05	.762
Dominant ABD	-.09	.560
Non-dominant Multifidi	.03	.839
Dominant Multifidi	.05	.765
**Contracted**		
Non-dominant EO	-.04	.785
Dominant EO	.23	.120
Non-dominant IO	-.11	.486
Dominant IO	-.26	.085
Non-dominant TA	.05	.737
Dominant TA	.18	.227
Non-dominant ABD	-.12	.430
Dominant ABD	-.05	.728

ϱ, Spearman's Rank order correlation performed for non-parametric data; EO, external oblique; IO, internal oblique; TA, transversus abdominis; ABD, abdominal wall

Supplementary material – Table 5. The relationship between bowling accuracy and derivatives of absolute muscle thickness

	ϱ	p-value
**Percentage difference**		
EO at rest	-.21	.163
IO at rest	-.06	.716
TA at rest	.16	.305
ABD at rest	-.02	.874
EO contracted	-.01	.969
IO contracted	.26	.085
TA contracted	.03	.845
ABD contracted	.00	.994
Multifidi	.36	.014
**Percentage change**		
Non-dominant EO	-.14	.364
Dominant EO	-.06	.699
Non-dominant IO	.10	.528
Dominant IO	-.12	.416
Dominant TA	.30	.046
Non-dominant TA	.14	.360
**Contraction ratio**		
Non-dominant EO	-.06	.699
Dominant EO	.10	.528
Non-dominant IO	-.12	.416
Dominant IO	-.14	.364
Non-dominant TA	.14	.360
Dominant TA	.30	.046
Non-dominant EOIO	-.10	.498
Dominant EOIO	-.02	.917
**Relative thickness at rest**		
Non-dominant EO	.06	.762
Non-dominant IO	-.05	.737
Non-dominant TA	-.06	.705
Dominant EO	.18	.243
Dominant IO	-.20	.182
Dominant TA	.02	.920
**Relative thickness contracted**		
Non-dominant EO	.00	.978
Non-dominant IO	-.09	.541
Non-dominant TA	.17	.271
Dominant EO	.27	.069
Dominant IO	-.37	.013
Dominant TA	.40	.006

ϱ, Spearman's Rank order correlation performed for non-parametric data; EO, external oblique; IO, internal oblique; TA, transversus abdominis; ABD, abdominal wall.
